# The Effect of Alternative Summary Statistics for Communicating Risk Reduction on Decisions about Taking Statins: A Randomized Trial

**DOI:** 10.1371/journal.pmed.1000134

**Published:** 2009-08-25

**Authors:** Cheryl L. L. Carling, Doris Tove Kristoffersen, Victor M. Montori, Jeph Herrin, Holger J. Schünemann, Shaun Treweek, Elie A. Akl, Andrew D. Oxman

**Affiliations:** 1 Norwegian Knowledge Centre for the Health Services, Oslo, Norway; 2 Knowledge and Encounter Research Unit, Division of Endocrinology and Internal Medicine, Mayo Clinic College of Medicine, Rochester, Minnesota, United States of America; 3 Flying Buttress Associates, Charlottesville, Virginia, United States of America; 4 Clinical Research and INFORMAtion Translation Unit and Department of Epidemiology, Italian National Cancer Institute Regina Elena, Rome, Italy; 5 Department of Medicine, University at Buffalo, Buffalo, New York, United States of America; Cardiff University, United Kingdom

## Abstract

**Background:**

While different ways of presenting treatment effects can affect health care decisions, little is known about which presentations best help people make decisions consistent with their own values. We compared six summary statistics for communicating coronary heart disease (CHD) risk reduction with statins: relative risk reduction and five absolute summary measures—absolute risk reduction, number needed to treat, event rates, tablets needed to take, and natural frequencies.

**Methods and Findings:**

We conducted a randomized trial to determine which presentation resulted in choices most consistent with participants' values. We recruited adult volunteers who participated through an interactive Web site. Participants rated the relative importance of outcomes using visual analogue scales (VAS). We then randomized participants to one of the six summary statistics and asked them to choose whether to take statins based on this information. We calculated a relative importance score (RIS) by subtracting the VAS scores for the downsides of taking statins from the VAS score for CHD. We used logistic regression to determine the association between participants' RIS and their choice. 2,978 participants completed the study. Relative risk reduction resulted in a 21% higher probability of choosing to take statins over all values of RIS compared to the absolute summary statistics. This corresponds to a number needed to treat (NNT) of 5; i.e., for every five participants shown the relative risk reduction one additional participant chose to take statins, compared to the other summary statistics. There were no significant differences among the absolute summary statistics in the association between RIS and participants' decisions whether to take statins. Natural frequencies were best understood (86% reported they understood them well or very well), and participants were most satisfied with this information.

**Conclusions:**

Presenting the benefits of taking statins as a relative risk reduction increases the likelihood of people accepting treatment compared to presenting absolute summary statistics, independent of the relative importance they attach to the consequences. Natural frequencies may be the most suitable summary statistic for presenting treatment effects, based on self-reported preference, understanding of and satisfaction with the information, and confidence in the decision.

**Clinical Trials Registration:**

ISRCTN85194921

*Please see later in the article for the Editors' Summary*

## Abstract 2

Carling and colleagues carry out a trial evaluating different methods of communicating information to people regarding the risks and benefits of taking statins. They suggest that natural frequencies are likely to be the most appropriate summary statistic for presenting the effects of treatment.

## Editors' Summary

**Background:**

People often have to make decisions about their health care. Ideally, all the health care decisions that a person makes should be those best suited to his or her personal circumstances and expectations. Take, for example, someone with a high amount of cholesterol (a type of fat) in his or her blood. Because this condition increases the chances of developing potentially fatal coronary heart disease (CHD), such a person will often be advised by his or her doctor to take statins to reduce blood cholesterol levels. However, the person needs to consider both the benefits and downsides of this course of action. Can he or she afford to pay for statins, if their health care system requires him or her to? Does the person want to take a pill every day that might cause some side effects? That is, the person has to consider his or her “values”—the relative desirability of all the possible outcomes of taking statins—before deciding whether to follow his or her doctor's advice.

**Why Was This Study Done?:**

It is well known that how information is presented to patients about treatment options and their consequences affects the choices that they make. For example, patients who are told that a drug will halve their chances of developing a disease (a 50% relative risk reduction) are more likely to decide to take that drug than those who are told it will reduce their absolute (actual) risk of developing the disease from 4% to 2%. Less is known, however, about which presentations of treatment effects best help people to make decisions that are consistent with their own values. In 2002, therefore, a series of internet-based randomized trials (studies in which participants are randomly allocated to different “treatment” groups) called the Health Information Project: Presentation Online (HIPPO) was initiated. Here, the researchers describe HIPPO 2, a trial that investigates how alternative summary statistics for communicating the reduction of CHD risk with statins affect people's decisions to take statins.

**What Did the Researchers Do and Find?:**

Nearly 3,000 adults in Norway and North America rated the relative importance to them of CHD risk reduction, the cost of statins, and the need to take a daily pill through an interactive Web site. The researchers used these data to calculate a “relative importance score” (RIS), an indicator of each participant's values. Each participant then decided whether or not to take statins after being shown one of six summary statistics about the effect of statins on CHD risk (relative risk reduction and five indicators of absolute risk reduction). The presentation of the effect of statins as a relative risk reduction resulted in more people deciding to take statins over the whole RIS range than any of the absolute summary statistics. For every five participants shown the relative risk reduction statistic, an extra participant chose to take statins compared to the other summary statistics. When asked to compare the six summary statistics, the statistic that most people preferred and understood best was the “natural frequency,” an absolute summary statistic that gave the number of people likely to develop CHD with and without statin treatment.

**What Do These Findings Mean?:**

Although these findings may not be generalizable to other populations or to other medical decisions, they provide new insights into how the presentation of information can affect the choices people make about health care. Specifically, these findings indicate that the presentation of the reduced risk of getting CHD as a result of taking stains as a relative amount is more likely to persuade people to take statins than several absolute summary statistics. They also suggest that the persuasive effect of the relative risk reduction summary statistic is not affected by the relative importance attached to the consequences of taking statins by individuals. That is, people shown the relative risk reduction statistic may be more likely to start statins to reduce their CHD risk (or a drug that reduces the risk of developing another disease) whatever their personal values than people shown absolute summary statistics. Finally, the findings on participant preferences suggest that natural frequencies may be the best summary statistic to include in tools designed to help people make decisions about their healthcare.

**Additional Information:**

Please access these Web sites via the online version of this summary at http://dx.doi.org/10.1371/journal.pmed.1000134.

- A *PLoS Medicine* Editorial discusses this trial and the results of another HIPPO trial that are presented in a separate *PLoS Medicine* Research Article by Carling and colleagues; details of a pilot HIPPO trial are also available
- The Foundation for Informed Medical Decision Making (a US-based non-profit organization) provides information on many aspects of medical decision making
- The Dartmouth-Hitchcock Medical Center provides information to help people make health care decisions through its Center for Shared Decision Making
- The Ottawa Hospital Research Institute provides also information on patient decision aids, including an inventory of decision aids available on the Web (in English and French)
- MedlinePlus provides links to information and advice about statins and about coronary heart disease (in English and Spanish)

## Introduction

For patients, health care professionals, and policy makers to make informed choices about health care, they must have information about the effects of interventions that is valid and understandable. The manner in which this information is presented affects choice [1],[2]. The goal of the Health Information Project: Presentation Online (HIPPO) is to evaluate alternative ways of presenting research evidence in order to improve communication of information about the effects of health care and to facilitate clinical decisions that are consistent with patient values. (“Values” here refers to the relative importance of the desirable and undesirable effects of an intervention.)

Systematic reviews [1],[2] have found that use of relative risk reduction (RRR) to represent the effect of treatment results in individuals perceiving a larger treatment effect and being more likely to decide in favor of treatment compared with the use of absolute risk reduction (ARR) or the number needed to treat (NNT) [1]. In studies to find a minimally important difference, ARR produced 20% larger differences in the medians than NNT (25% versus 5%). The same review found that presenting the percentage of people experiencing outcomes with and without treatment resulted in more accurate perceptions than RRR when baseline risk was also provided. Others have advocated summary measures that have been less well studied, such as the tablets needed to take (TNT) [3] and “natural frequencies” (NF), i.e., raw observations that have not been transformed into percentages. Gigerenzer and others [4]–[7] advocate this format as a way of facilitating correct decisions, especially in diagnostics, among physicians as well as lay people. Hollnagel proposed a similar summary statistic, referred to as whole numbers [8].

There is a large literature that addresses how different presentations, including positive versus negative framing, different summary statistics, and different formats (numeric, verbal, or graphical) influence understanding, perceptions, and decisions; and how information about risk is used in decisions [1]–[16]. However, to our knowledge, there are no studies investigating the relationship between the summary statistic used and the extent to which decisions are consistent with individuals' values, apart from our pilot study [17].

Thus, we designed this study to assess the extent to which the use of different summary statistics affects choices about using statins to reduce the risk of coronary heart disease (CHD). We chose this decision because it is common, important, familiar to many, and because high-quality evidence is available about the benefits and downsides of statins [18]. It is a “preference sensitive” decision that is affected by patients' values [19]. Thus, among people with the same hypothetical or real risk of CHD, one would expect some degree of correlation between how important the desirable and undesirable consequences of taking statins are to them and the likelihood that they would decide to take statins. In other words, one would expect that people for whom the benefits of taking statins were less important and the downsides more important would be less likely, on average, to decide to take them than people for whom the benefits were more important and the downsides were less important.

## Methods

### Study Design

The CONSORT checklist and the protocol for this study are available as supporting information; see Text S1 and S2.

This study was an Internet-based randomized trial (Text S3) in which participants were randomized to one of six summary statistics presenting information about CHD risk reduction associated with statin use (Text S4). A pilot study of similar design with 770 participants in 2002 informed the final design of this trial [17]. Data from the pilot study are not included in this report.

### Recruitment, Eligibility, and Allocation

The study and its Web site link were advertised to the public in Norway and North America through traditional media (radio, TV, flyers in public spaces including physician offices) and through online ads in Web portals and health-related Web sites. To encourage participation, we offered prospective participants the option to take part in a lottery for a $100 gift certificate and to receive study results.

Only complete responses from people who reported being 18 years or older, and that they were answering for the first time, were included in the analysis.

We randomized participants to one of the six presentations upon log-on by block-randomization (Text S4), using a looped sequence of 600 presentation assignments consisting of 100 blocks of six that was generated on http://www.randomization.com.

### Data Collection

**Figure 1:**
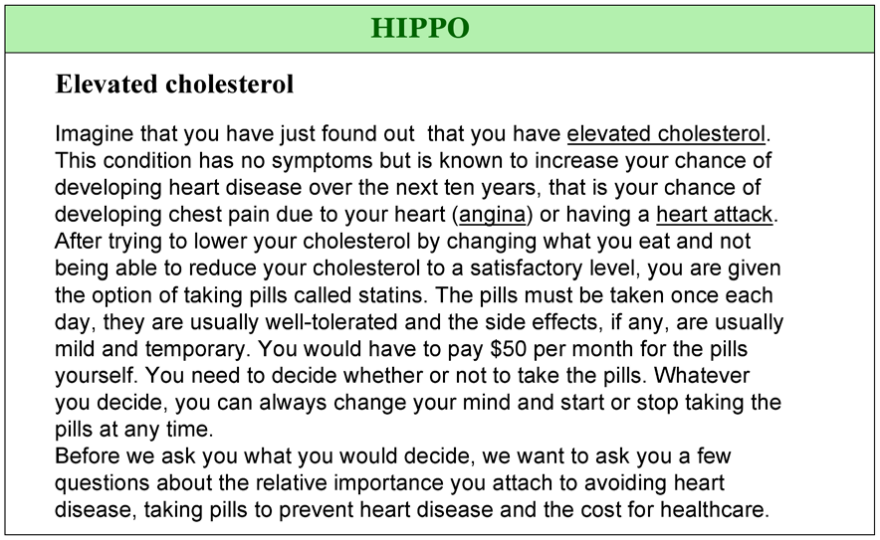
Introduction to the scenario prior to eliciting values.

After choosing to log onto either the Norwegian or English-language version of the study Web site, participants received information about the study and gave informed consent to participate. Then, participants reviewed a hypothetical scenario involving themselves as patients with elevated cholesterol whose doctors had offered them the option of taking pills (statins) to lower blood cholesterol levels to reduce their risk of CHD (Figure 1). They learned that they had to take a pill each day and incur an out-of-pocket cost for the pills of US $50 (400 NOK) per month.

Then participants reported the relative importance they assigned to getting CHD, to a monthly increase of US $50 in health care costs, and to having to take a pill every day, using a horizontal 100-point visual analogue scale (VAS) with “No problem” and “Very difficult” as the lower and upper anchors.

**Figure 2:**
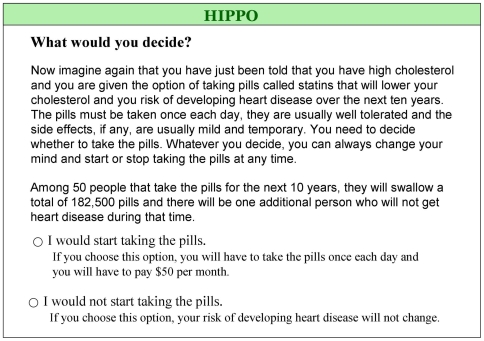
One of six presentations to which participants were randomized after eliciting values.

**Figure 3:**
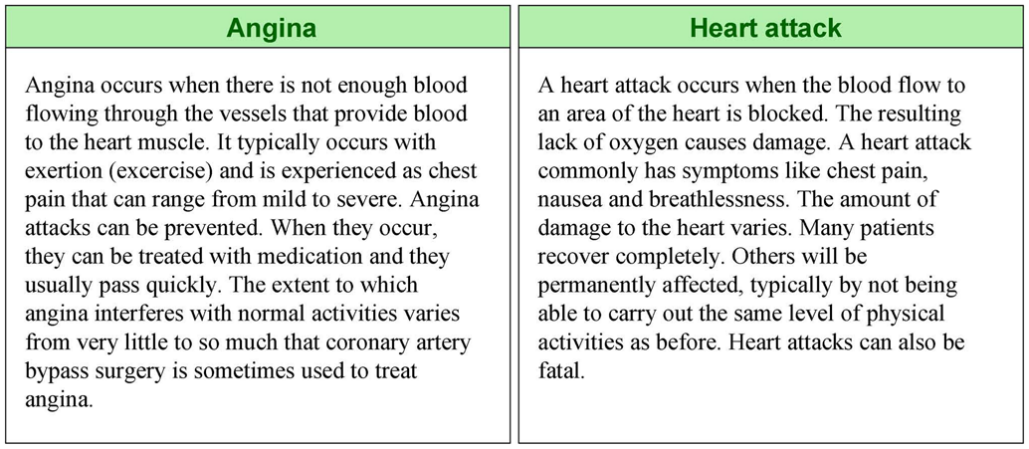
Examples of hypertext links describing a heart attack and angina.

Participants then considered the effectiveness of statins in reducing the risk of CHD using their randomly assigned summary statistic (Figure 2). The summary statistics reflected a 10-year CHD risk of 6% without statins (estimated risk for a person without other risk factors than a high cholesterol level [20]) and the 30% relative reduction in the risk of CHD with statins [21]. Participants then had to decide whether or not to take statins. They could access additional explanations of terms such as “angina” and “heart attack” using hyperlinks in the text (Figure 3) or navigate back to previously answered screens and change their answers, but could not return after having made a decision.

**Figure 4:**
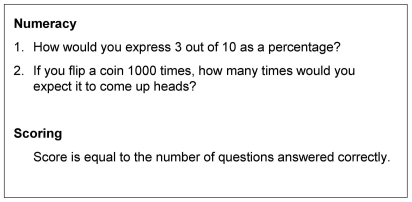
Numeracy assessment.

**Figure 5:**
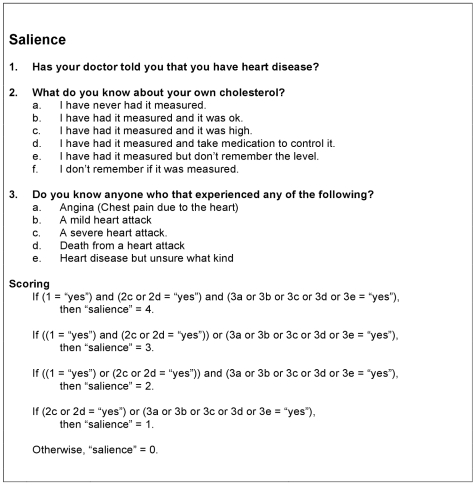
Salience questionnaire.

After making this decision, participants reported, using a 5-point scale where 5 was the highest rating, on their confidence in their decision and understanding of and satisfaction with the information. Participants then completed a numeracy assessment (Figure 4), a salience questionnaire (to measure how relevant or important the hypothetical scenario was likely to be to the participants) (Figure 5), and reported sociodemographic data. Then, they reviewed all six summary statistics and were asked which one they preferred.

### Analysis

We calculated a relative importance score (RIS) for each participant by subtracting from her VAS score for CHD the sum of her VAS scores for increased health care costs and having to take a pill every day. We expected that higher RIS would be correlated with an increased likelihood of deciding to start taking statins.

The trial sought to test, in terms of concordance between decisions and RIS values, the following three hypotheses, based on the results of a pilot study [17]:

1. RRR results in a higher likelihood of deciding to start taking statins across RIS values compared to the absolute summary statistics.
2. The slope of the log odds of ARR is greater than the slope of the other absolute summary statistics.
3. The concordance between decisions and values for the event rate (ER) is less than for the other absolute summary statistics; i.e. that the slope for the relationship between RIS values and the log odds of deciding to take statins is not significantly different from zero for ER (indicating that decisions were independent of the participants' elicited values), whereas it is positive (consistent with what would be predicted) and significantly different from zero for the other absolute summary statistics.

In order to evaluate the effects of the different summary statistics on the decision to start statins, taking into account each participant's RIS, we used the following logistic regression model:
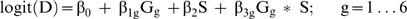
where D is the decision to go to take statins or not, G is the presentation group, S is the RIS value, and G*S is the interaction between the presentation and the RIS value. To make inferences about the response within each group and for the comparisons of groups we used dummy variable coding with reference parameterization for the presentation groups, i.e., directly estimating the difference in the effect of each nonreference level compared to the effect of the reference level. We used Wald tests to compare the *p*-values and confidence intervals (CIs) from the logistic regression and Chi-square tests to compare frequenices. The model was also explored by including numeracy and salience as covariates.

We used the model without covariates to test the three hypotheses based on the results of our pilot study [17] by (1) comparing the log odds of RRR to the log odds of the pooled absolute summary statistics for the RIS value at which the predicted log odds was zero (i.e. odds = 1) for the pooled summary statistics; (2) comparing the slope of the log odds of ARR to the slope of the pooled absolute summary statistics (excluding ARR), and (3) comparing the slope for the ER group to zero and testing for a difference in slope between ER and the slope of the pooled summary statistics excluding ER. Thus, we used a Bonferroni correction for four comparisons to adjust the confidence intervals for these analyses corresponding to an overall significance level of 0.05 (i.e. 0.05/4 = 0.0125) although the sample size estimates were based on three comparisons.

We performed additional comparisons for the difference in log odds at the median, 1st, and 3rd quartile values for RIS, and we compared the slope for the ER group to the slope for the pooled absolute summary statistics group (excluding ER) We did not adjust the 95% CIs or *p*-values for these or other comparisons. They should be interpreted with caution due to multiple testing since no assessment of their power was made in the protocol.

### Sample Size

Using data from the pilot study, we estimated we would need about 750 to 800 participants per group to achieve 80% power to test each one of the three hypotheses at 0.0167 alpha level (after applying a Bonferroni correction).

### Ethics

The protocol was reviewed and approved by the ethics committee of the University at Buffalo Medical School, Buffalo, New York. Participants gave informed consent via the Web site interface, having been given information about the study and told that they could quit at any time and request that their data be deleted. Contact information that some participants supplied in order to request the study results or to participate in the lottery was stored in a separate database that was not linkable with study data.

## Results

The study recruited 2,978 eligible participants between June 2003 and July 2005 (Table 1). We decided to stop recruitment prior to achieving the intended sample size after multiple efforts to encourage participation over two years. We did not look at the results prior to deciding to stop the study. The six groups were similar, with respect to sex, age, years of education, salience of the scenario, and their elicited values. Most respondents were from USA (42%) and Norway (26%). Seventy-three percent of the participants chose the English language version of the Web site. Numeracy varied across the six groups from 65% that answered both questions correctly in the RRR group to 75% in the TNT and NF groups.

**Table 1:** Participant characteristics.

Category	Subcategory	RRR	ARR	NNT	ER	TNT	NF	Overall
		*n* = 508	*n* = 505	*n* = 484	*n* = 476	*n* = 512	*n* = 493	*n* = 2,978
**Women** a		58.3	58.8	62.6	61.6	56.8	56.8	59.1
**Age** a	18–39	37.2	35.5	36.5	32.5	38.9	36.3	36.2
	40–59	53.3	53.9	54.3	56.9	50.6	51.9	53.5
	≥60	9.5	10.7	9.1	10.5	10.5	11.7	10.4
**Years of education** a	≤8 y	1.6	2.2	1.7	1.9	2.1	2.4	2.0
	9–12 y	7.3	7.9	5.6	5.7	6.4	6.5	6.6
	13–16 y	27.6	26.9	30.2	28.6	28.5	29.8	28.6
	≥17 y	63.6	63.0	62.6	63.9	62.9	61.3	62.9
**Country of residence** a	Canada	15	12	13	15	10	13	12.4
	Germany	1	1	1	0	0	0	0.6
	Norway	29	26	29	23	25	30	26.5
	US	40	36	38	43	42	40	41.5
	Other	15	24	19	18	22	18	19.0
**Language** a	English	72	74	71	78	74	71	73.4
	Norwegian	28	26	29	22	26	29	26.6
**Numeracy** a	2 correct answers	64.6	67.7	73.1	71.6	75.0	74.8	71.1
	1 correct answer	30.1	28.1	22.1	23.5	20.1	20.5	24.1
	0 correct answers	5.3	4.2	4.8	4.8	4.9	4.7	4.8
**Salience score** a	4 (high salience)	4.5	5.0	4.3	3.4	5.5	3.9	4.4
	3	2.4	1.4	2.7	1.5	2.0	2.8	2.1
	2	25.4	25.1	24.4	27.7	23.4	26.0	25.3
	1 (low salience)	60.8	61.8	65.5	62.2	63.5	60.4	62.4
	0 (no salience)	6.9	6.7	3.1	5.3	5.7	6.9	5.8
**Values (on 100-point VAS)** b	CHD	80.7 (21)	78.5 (23)	79.7 (21)	80.1 (21)	79 (21.8)	78.8 (23)	79.5 (22)
	Cost	30.2 (26)	32.1 (28)	33.3 (27)	31.1 (27)	31 (27)	29.7 (27)	31.2 (27)
	Pill taking	21.4 (23)	24.3 (26)	23.4 (26)	22.1 (24)	24.2 (24)	23.3 (25)	23.1 (25)
	RIS	29.1 (44)	22.2 (49)	23 (47)	27 (48)	23.8 (48)	25.7 (47)	25.1 (47)

a Data presented as percentages of *n* in a given column.

b Data presented as mean (standard deviation) for a given presentation group.

The most common ways in which participants reported finding out about the study were a link on another Web site (32%), an email invitation (27%), and a link to the study sent by a friend (18%). Fifty-nine percent of the participants were women, 54% were between 40 and 59 years old, and 63% had 17 or more years of education. Seventy-one percent answered both questions assessing numeracy correctly, and the scenario had low or no salience for 68%, based on their experience with CHD and hypercholesterolemia.

The participants' preferred presentation was natural frequencies (31%) closely followed by RRR (30%). Event rates were preferred by 20%, NNT by 10%, ARR by 5%, and TNT by 3%.

### Decisions in Relation to Values

There was a significantly larger proportion in the RRR group that decided to start taking statins (74%) than in the “absolute” summary statistics groups (range 51% to 56%) (Table 2).

**Table 2:** Decisional outcomes.

Outcome	RRR	ARR	NNT	ER	TNT	NF	Overall	*p-*Value^a^
**Decided to start taking statins**, *n* (%)	376 (74)	256 (51)	267 (55)	252 (53)	284 (55)	256 (52)	1,691 (57)	<0.001
**Understanding of information**, median (IQR)	4 (4,5)	4 (3, 5)	4 (4,5)	4 (4,5)	4 (4,5)	5 (4,5)	4 (4,5)	
High rate (4 or 5 out of 5), *n* (%)	394 (78)	368 (73)	369 (76)	272 (78)	386 (75)	422 (86)	2,310 (78)	0.002
**Satisfaction with information**, median (IQR)	3 (2, 4)	3 (2, 4)	3 (2, 4)	3 (2, 4)	3 (2, 4)	3 (2, 4)	3 (2, 4)	
High rate (4 or 5 out of 5), *n* (%)	163 (32)	158 (31)	157 (32)	150 (32)	145 (28)	201 (41)	974 (33)	0.001
**Confidence in decision**, median (IQR)	4 (3, 5)	4 (3, 4)	4 (3, 4)	4 (3, 4)	4 (3, 4)	4 (3, 4)	4 (3, 4)	
High rate (4 or 5 out of 5), *n* (%)	317 (62)	287 (57)	270 (56)	282 (60)	302 (62)	305 (62)	1,763 (59)	0.62

a *p*-Values from Chi-square tests without corrections for multiple testing.

IQR, interquartile range.

**Figure 6:**
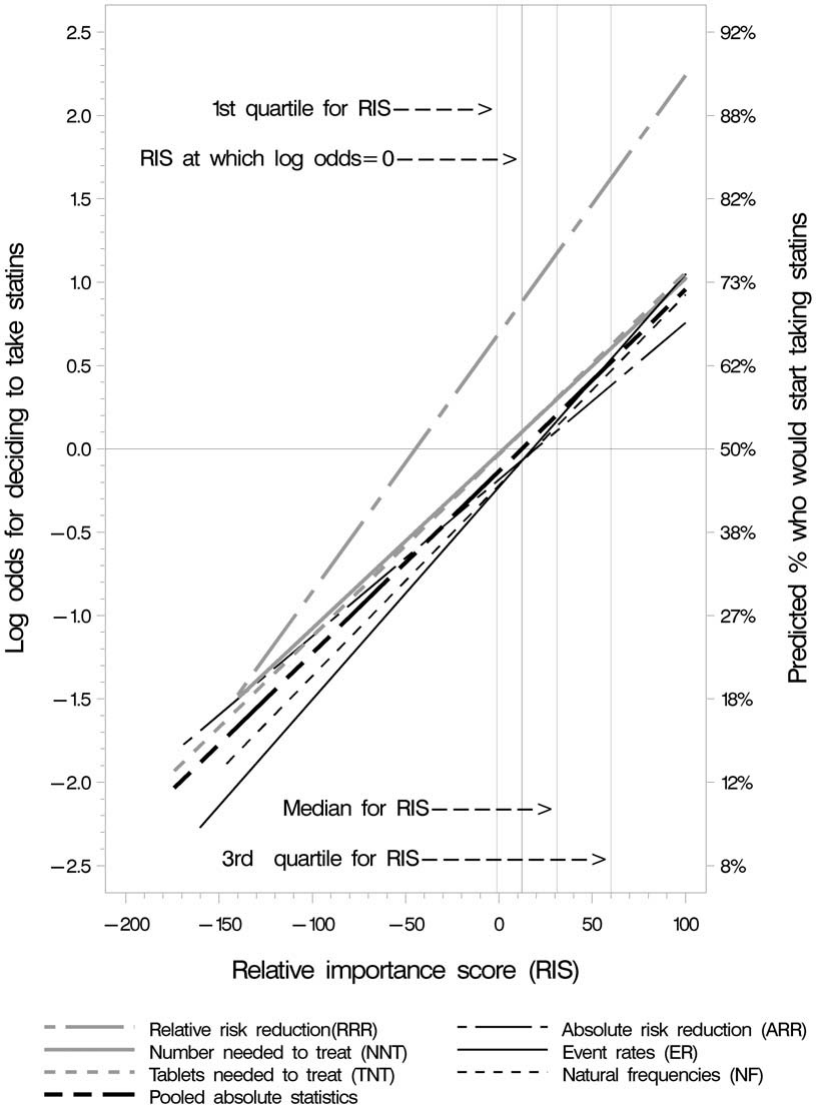
Likelihood of deciding to start taking statins in relation to RIS.

Participants in all groups were less likely to take statins when the relative importance score (RIS) was lower and more likely when it was higher (Figure 6). There were no differences in the association between RIS and the likelihood of taking statins across the five absolute summary statistics, including event rates (Table 3).

**Table 3:** Likelihood of deciding to take statins in relation to values (RIS).

Presentation Group	Slope (beta) (95% CI)	1^st^ quartile RIS = −1		Median RIS = 31		3^rd^ quartile RIS = 60	
		Odds (95% CI)	Predicted % “take”	Odds (95% CI)	Predicted % “take”	Odds (95% CI)	Predicted % “take”
**RRR**	0.015 (0.011–0.020)	1.97 (1.57–2.47)	66.3 (61.0–71.2)	3.23 (2.60–4.00)	76.3 (72.2–80.0)	5.06 (3.80–6.73)	83.5 (79.2–87.1)
**ARR**	0.009 (0.006–0.013)	0.82 (0.67–1.01)	45.2 (40.2–50.2)	1.11 (0.93–1.33)	52.7 (48.1–57.2)	1.46 (1.16–1.84)	59.4 (53.8–64.8)
**NNT**	0.010 (0.006–0.015)	0.96 (0.78–1.18)	49.1 (43.9–54.2)	1.35 (1.12–1.63)	57.4 (52.8–61.9)	1.83 (1.44–2.32)	64.6 (59.0–69.9)
**ER**	0.013 (0.007–0.018)^a^	0.79 (0.63–0.98)	44.0 (38.6–49.6)	1.18 (0.98–1.43)	54.2 (49.5–58.8)	1.71 (1.36–2.16)	63.1 (57.6–68.3)
**TNT**	0.011 (0.007–0.015)	0.96 (0.78–1.17)	48.9 (43.8–54.0)	1.36 (1.13–1.63)	57.5 (53.0–61.9)	1.86 (1.47–2.34)	65.0 (59.6–70.1)
**NF**	0.011 (0.007–0.016)	0.79 (0.64–0.99)	44.3 (39.0–49.6)	1.15 (0.95–1.38)	53.4 (48.8–57.9)	1.60 (1.27–2.01)	61.5 (55.9–66.7)
**Pooled absolute statistics**	0.011 (0.009–0.013)	0.86 (0.79–0.95)	46.4 (44.0–48.7)	1.23 (1.13–1.33)	55.1 (53.0–57.1)	1.68 (1.52–1.87)	62.7 (60.3–65.1)
**Pooled absolute statistics except ARR**	0.011 (0.009–0.013)	0.87 (0.79–0.97)	46.7 (44.0–49.3)	1.26 (1.15–1.38)	55.7 (53.4–57.9)	1.74 (1.55–1.96)	63.6 (60.8–66.2)

Predicted % = proportion deciding to take antihypertensive medication based on logistic regression.

a Alpha = 0.0125.

The testing of the three main hypotheses gave the following results:

1. The difference in log odds of RRR versus the pooled absolute summary statistics at RIS = 12.3 (the point at which the predicted log odds was zero [odds = 1] for the pooled absolute statistics) was 0.88 (*p*<0.0001; odds ratio = 2.4; adjusted 95% CI 1.8 to 3.2). The odds of the RRR group choosing to take statins was two to three times greater at the median and the 1^st^ and 3^rd^ quartiles (Table 4) corresponding to differences of 20% to 21% in the proportions of participants choosing to take statins (Table 3).
2. The difference in the slope of the estimated log odds of ARR to the slope of the pooled absolute summary statistics (excluding ARR) was 0 (*p* = 0.4, adjusted 95% CI −0.008 to 0.004).
3. The slope of the log odds for the ER group was statistically significantly different from zero (β = 0.013, adjusted 95% CI 0.007 to 0.018) (Table 3), as it also was for the slope for the pooled absolute summary statistics group excluding ER (β = 0.0105, adjusted 95% CI 0.009 to 0.013). There was no significant difference between them: point estimate 0 (*p* = 0.3, adjusted 95% CI −0.004 to 0.008).

**Table 4:** Comparisons.

	1^st^ quartile RIS = −1	Median RIS = 31	3^rd^ quartile RIS = 60
**RRR versus pooled absolute statistics**	2.27 (1.78–2.91)	2.63 (2.09–3.32)	3.00 (2.22–4.07)
**ARR versus pooled absolute statistics except ARR**	0.94 (0.75–1.18)	0.89 (0.72–1.09)	0.84 (0. – 1.08)

Data are given as odds ratio (95% CI).

There were few responses for the salience categories of 0, 3, and 4, and few responders with no correct numeracy answers. We therefore pooled and renamed the categories of salience: “Low salience (0, 1),” “Some or high salience (2, 3, and 4)”; and numeracy: “Low numeracy (0, 1)” and “Correct (2)” and entered these covariates into the model. Salience was nonsignificant (*p* = 0.4). Although numeracy was significant (*p* = 0.01), it had a minor impact on the effect estimates and the standard error. The *c*-statistic for the overall model including covariates compared to the model without covariates was the same (*c* = 0.671).

### Understanding and Satisfaction

In terms of understanding, 86% reported they understood NFs well or very well (Table 2). Confidence in the decision was not different across randomized groups (*p* = 0.62). Across all participants, confidence in the decision was associated with the decision to start statins (*p*<0.0001). Among those deciding to take statins 65% scored their confidence as 4 or 5 (anchored as “extremely confident” at a score of 5) compared to 51% among those who decided not to take statins.

The NF group was the most satisfied with the risk information that they had initially received (41% rated their satisfaction as 4 or 5 anchored as “extremely satisfied” at a score of 5); least satisfied were the TNT and the ARR groups (28% rated their satisfaction as 4 or 5) (Table 2). Across all participants, satisfaction in the risk information was associated with deciding to take statins (*p*<0.001).

## Discussion

There was a clear relationship between participants' summary relative value scores expressed as RIS and the choices that they made across all summary statistics; i.e., as RIS increased, the likelihood of choosing to start statins increased, as would be expected for a preference sensitive decision. RRR resulted in the largest proportion of participants deciding to start statins compared to the absolute summary statistics. Participants in the RRR group were more likely to decide to start statins at all values of RIS than participants in the absolute summary statistic groups. The increased probability to choose to start statins in the RRR group is consistent with conclusions from other studies and supports the contention that RRR is a more persuasive summary statistic [1],[2],[13]–[16].

This is the first study of which we are aware that shows that people are more likely to be persuaded when presented with a relative summary statistic regardless of their values. The findings of this study confirm the results of our pilot study [17] with respect to RRR resulting in participants being more likely to decide to take statins regardless of their values. The findings did not support either of the two other hypotheses generated from the results of our pilot study. ARR did not result in a steeper slope of the estimate of the likelihood (log odds) of deciding to take statins relative to participants' RIS values. Event rates did not result in decisions disassociated from values and, in fact, produced an estimate that did not differ from the other absolute summary statistics.

In contrast to the pilot, where the majority (52%) preferred RRR, only 30% preferred RRR in this study and NF were preferred by 31% (compared to 25% in the pilot). In both studies, few participants preferred TNT (3% and 1% in this and the pilot study respectively) and ARR (5% and 4%). NNT was preferred by more participants in this study (10%) than in the pilot (4%).

Despite a 2-year recruitment period, the estimated sample size requirement was not met. This problem of recruiting participants to Internet-based studies has been reported elsewhere [22]. However, randomization worked well, generating six comparable groups.

### Applicability of the Findings

We used a variety of strategies to recruit a convenience sample of participants, including placing links on other Web sites, sending email invitations, encouraging people to send the link to friends, and sending messages to discussion lists. Therefore, there is not a sampling frame from which participants were drawn to which we can compare them. The participants, however, had a relatively high level of education and it is uncertain that the findings are applicable to populations with less education [2],[17]. It is also uncertain to what extent results from the hypothetical scenario used in this study apply to actual decisions [13],[23], to personal communication [13], to other populations, or to decisions with higher risk levels.

While the results of Internet-based studies such as this one likely apply to printed as well as electronic information, they may not apply to personal communication, when it is possible to interact and adapt the presentation of information. The influence of how information is presented on decision making may also vary in relation to the salience of the scenario to decision makers [17],[24] and to their level of numeracy [17],[25], although neither significantly modified our results (data not shown).

### Conclusions

Presentation of the RRR increases the likelihood of people accepting treatment over that of absolute summary statistics, independent of the relative importance they attach to the consequences. We did not find important differences in the relationship between decisions and values among the five absolute summary statistics we tested. However, natural frequencies may be preferable, based on self-reported preference, understanding and satisfaction with the information, and confidence in decision. This result supports the advice of others that natural frequencies should be the preferred summary statistic for decision aids and other risk communication tools [4]–[7].

## Supporting Information

Text S1 CONSORT checklist.
(0.04 MB PDF)

Text S2 Study protocol.
(0.15 MB DOC)

Text S3 CONSORT flow chart.
(0.01 MB PDF)

Text S4 Risk presentation.
(0.01 MB PDF)

## Abbreviations

ARR: absolute risk reduction
CHD: coronary heart disease
ER: event rate
NF: natural frequency
NNT: number needed to treat
RIS: relative importance score(s)
RRR: relative risk reduction
TNT: tablets needed to treat
VAS: visual analogue scale(s)
