# Supplementary material for: The Effect of Alternative Summary Statistics for Communicating Risk Reduction on Decisions about Taking Statins: A Randomized Trial
Source: PLoS Med. 2009 Aug 25;6(8):e1000134. doi: 10.1371/journal.pmed.1000134 (PMC2724738; doi:10.1371/journal.pmed.1000134)
Supplement: Text S2 — Study protocol. (0.15 MB DOC) [file pmed.1000134.s002.doc]

**The effect of alternative summary statistics for communicating risk reduction on decisions about taking statins: A randomized trial**

PROTOCOL

Cheryl Carling, RN, MSc, Research Fellow^1^

Holger Schünemann, MD, PhD, Assistant Professor^2,3^

Jeph Herrin, PhD, Research Consultant^4^

Jan Arve Dyrnes, Consultant^1^

Shaun Treweek, PhD, Researcher^1^

Doris Tove Kristoffersen, MSc, Researcher^1^

Elie Akl, MD, Resident^2^

Phillip J. Deveraux, MD, Research Fellow^3^

Victor Montori, MD, MSc^3^

Andrew David Oxman, MD, Director^1^

1. The Norwegian Knowledge Centre for the Health Services

P.O. Box 7004 St. Olavs plass

N-0130 Oslo

Norway

2. Departments of Medicine and Social and Preventive Medicine

University of Buffalo

270 Farber Hall

3435 Main St

Buffalo, NY 14214

USA

3. Department of Clinical Epidemiology and Biostatistics

McMaster University

Room 2C12

Hamilton, Ontario, L8N 3Z5

Canada

4. Flying Butress Associates

PO Box 2254

Charlottesville, VA 22902

USA

Address for correspondence:

Cheryl Carling

The Norwegian Knowledge Centre for the Health Services

P.O. Box 7004 St. Olavs plass

N-0130 Oslo

Norway

# BACKGROUND

For patients, healthcare professionals and policy makers to make informed choices about healthcare, they must have information about the effects of interventions that is valid and understandable. The manner in which this information is presented affects how it is understood and subsequent decisions^1,2^. The objective of the Health Information Project: Presentation Online (HIPPO) is to improve communication of information about the effects of healthcare based on randomised trials of alternative ways of presenting this evidence, facilitating decisions that are consistent with personal values.

This is the first HIPPO trial. It is informed by results of a pilot study conducted October – November 2002 comprising 770 participants ^3^. Information about the effects of drugs used to decrease cholesterol blood levels was chosen for this study because it is a common problem and high quality evidence is available on the effects of cholesterol lowering drugs (statins).^4^ The results of this study will help to inform decisions about how best to communicate the benefits of statins to healthcare professionals, individual patients and the general public.

Two systematic reviews have concluded that the use of "relative risk reduction" (RRR) to express the relationship between two probabilities results in individuals having a larger estimate of that relationship and being more likely to decide in favour of treatment compared with the use of the absolute risk reduction (ARR) or the number needed to treat (NNT). ^1,2^ None of the studies included in these reviews has investigated the relationship between the summary statistic that is used and the extent to which decisions are congruent with peoples' values The results of our pilot study suggest that the RRR resulted in a disproportionately higher propensity for participants to choose pills, regardless of their elicited values.^3^

According to Decision Analysis, based on the normative concept of expected utility maximization,^5^ people should choose from among options the one which gives the highest expected utility, i.e. value. Expected utility for any choice is calculated by multiplying the probability for each expected outcome by the utility for that outcome, and summing the products for each outcome under that choice option.

EU = Σ p_i_ * u_i_

i

The utility of outcomes, such as different health states, is usually expressed as being between zero and one (e.g. with death having a value of zero and a fully healthy life having a value of one).

When analyzing the pilot data, alternative models of weighting the participants' elicited relative values for the outcomes associated with high cholesterol and taking statins for this indicated that an unweighted linear model worked as well as the expected utility model. Therefore, in the analysis of this study, probability will be eliminated from the model and the participant value of health states (PV) will be calculated by summation of the elicited relative values.

# OBJECTIVES

The objective of this study is to determine which of 6 ways of presenting information (Table1) about the reduction in risk of cardiovascular disease (CVD) associated with statin use results in decisions that are most congruent with what individuals considering starting statins for the primary prevention of CVD would be expected to decide based on their elicited relative values. Based on the results from the pilot study, we have three main hypotheses:

1. Is the congruence between participants’ values and their treatment choice less for the RRR as compared to the other summary statistics used in this trial?
2. Does the ARR result in higher congruence between participants’ values and their treatment choice than the other summary statistics used?
3. Does it appear that the event rates (ERS) results in random association between participants’ utilities and their treatment choice?

# METHODS

# Study design

The study is a Web-based randomised trial in which participants are randomised to one of six ways of presenting information about CVD risk reduction. (table 1)

We have chosen four of the presentations (RRR, ARR, NNT and ERS) based on conclusions from a Cochrane review.^2^ In comparisons of RRR and ARR, it was found that individuals perceived effects stated as RRR to be greater that those stated as ARR. Studies comparing RRR with NNT found RRR to be significantly more persuasive. In studies comparing ARR with NNT, there was inconclusive evidence as to persuasiveness. In studies to find Minimally Important Difference, ARR produced 20% larger differences in the medians than NNT (25% vs. 5%). Also, RRR was shown to be more persuasive in comparisons with ARR, NNT, and PEF (percent event free patients). Event rates (ERS) is the presentation of ARR with baseline risk provided. The same review showed that this resulted in more accurate perceptions than RRR when baseline was also provided. The construction of Tablets-needed-to-take (TNT) was proposed by Skolbekken^6^ and the Whole Numbers (WNUM) construction was proposed by Hollnagel.^7^

Upon logging into the study Website, participants will be presented with information about the study and asked to give informed consent to participate. They will view a brief scenario in which they will be asked to imagine that they have elevated cholesterol and that their doctor has given them the option of taking pills that lower cholesterol blood levels (statins). Each participant's relative values for getting CVD, taking pills and cost associated with healthcare will be elicited by using a visual analogue scale (VAS). Participants will then be randomly presented with one of the 6 presentation formats about the effect of statins on lowering the baseline risk of CVD and asked whether they would decide to take the pills or not. Baseline risk will then be altered in the direction that shifts the participant's values more in favour of the opposite decision, i.e. the baseline risk will be increased for participants who decide not to take the pills and decreased for those who decide to take the pills (table 2). Thereafter, the decision not to take or to take statins will be elicited again. Finally, participants will be asked questions about their decisions, about themselves and their preferred presentation format among the 6.

Inclusion criteria

Only complete responses from persons who where at least 18 years old and literate in the language that they selected to participate in the study will be included in the analysis.

Participants will be recruited in Norway by a media campaign on Webportals and television, through a physicians' online discussion group, and will be approached in public places such as doctors' offices, shopping malls and libraries. In the USA and Canada, we will approach patients via links on health related websites, email messages to primary care providers, public radio campaigns, department grand rounds, and in patient waiting rooms. In addition we will place advertisement notes that provide links to the website in physician offices and university buildings. Potential participants will be offered options to participate in a lottery for a $100 gift certificate and to receive a report of study results.

Allocation

Allocation to one of the 6 presentations is determined by block-randomization. We created a sequence of 600 presentation assignments by generating 100 blocks of the 6 presentations using [http://www.randomization.com](http://www.randomization.com/) (Randomization plan created on 17 April 2002, 16:03:41. To reproduce this plan, use the seed 11049). This sequence will be looped and repeated.

Data collection

Participants enter responses directly by responding to the questions on the HIPPO website. The data generated will be stored anonymously in a database. Only completed records from participants answering for the first time will be used in the main analyses.

Analysis

The results from the pilot study indicated that various methods of weighting the elicited relative values for outcomes related to high cholesterol and taking statins only minimally affected the value for a participant's value of a health state PV.^3^. Thus, in this study, a participant's value of health states PV is given by an unweighted linear model, using the elicited relative values, i.e. the VAS scores:

R = VAS_Pill_  + VAS_Cost_ - VAS_CVD_

The main objective of the study will be to identify which of the six summary statistics helps participants to make the decision to take or not take statins that is most consistent with what individuals would be expected to decide based on their ~~elicited values~~ PV. We expect individuals having a high PV to decide not to take statins, and persons with a low PV to start taking statins. That is, the probability for taking pills should be fairly high for persons who rank their importance of having heart disease much higher than the sum of their other values, whereas having more concern about having to pay for the pills and taking pills should be reflected in a very low probability.

The data will be analysed using a binary logistic regression model to identify which of the summary statistics result in the above. If G is used to represent the presentation group to which a participant is randomised and D represents the decision to take pills or not, then the relationship between D, G and R, can be modelled as follows:

(Eq 1)          logit(D) = β_0_ + β_1g_G_g_ + β_2_R + β_3g_G_g_*R; g=1,..,6,

If the decision can be explained by the summary statistics, R will be a significant variable in the model. A difference between the various summary statistics can be evaluated by comparison of the β_1g_s and β_3g_s (if an interaction is present). The group providing the steepest slope of the linear predictor might identified as the group providing the best congruence between PV and their treatment choice. This, however, will not be the most reliable result for a group if it has a very high proportion of individuals deciding to take pills and those who decide not to take pills have a fairly low PV. The slope coefficient gives the change in the log odds for an increase of 1 unit in R, and the difference in slope between two groups is the log odds ratio if the two groups have the same distribution of participant values. The odds equals one when the proportion deciding to take pills equals the proportion deciding not to take pills. The value of R for which this occurs will be an obvious value for comparison of the summary statistics groups, in addition to the slope comparison.

The results from the pilot study indicated that the linear predictors for the NNT, TNT and WNUM are similar with respect to both slope and location and thus can be pooled. The following hypotheses were established from the pilot trial:

1. There is no difference in the values of the linear predictor between the RRR group and the pooled estimate of the other groups at the total utility value for which the value of the linear predictor equals zero for the pooled group, versus the alternative hypothesis claiming a difference.
2. There is no difference in slope of the ARR group compared to the slope estimated from the pooled data for the other groups not including the RRR group, versus the alternative hypothesis claiming a difference.
3. there is no difference in slope of the ER group compared to estimate based on the pooled data for the other groups not including the RRR group versus the alternative claiming a difference.

If the data from this trial turns out to be inconsistent with the assumptions for pooling groups, the pooling might have to be reconsidered for the analysis.

Sample size

Sample size calculation is based on the estimates obtained from the logistic regression of the pilot-study data. For each group, the log odds for the decision to take pills versus not to take pills was calculated as a function of the patient’s relative importance. A power of about 80% for each of the tests is needed in order to reject the null hypotheses. To ensure an overall level of 0.05 significance, the level of significance for each test is 0.05/3 = 0.0167 (according to Bonferroni correction).

The following table shows the power for various numbers of subjects in each group when testing the first hypothesis i).

|  | **α=0.05** | | | | **α=0.017** | | | |
| --- | --- | --- | --- | --- | --- | --- | --- | --- |
| **ForContrast** | **Power, n=50** | **Power, n=60** | **Power, n=70** | **Power, n=80** | **Power, n=50** | **Power, n=60** | **Power, n=70** | **Power, n=80** |
| RRR vs ALL, disutility=-47  Difference=1.123 | 0.77473 | 0.81303 | 0.88749 | 0.90822 | 0.62846 | 0.67810 | 0.78422 | 0.81677 |

When comparing the slopes, more patients are needed if the differences are small. The table below shows the power for various numbers of subjects for the two other main hypotheses.

|  | **α=0.05** | | **α=0.017** | |
| --- | --- | --- | --- | --- |
| **Contrast** | **power** | **n** | **power** | **n** |
| ARR vs WNUM+TNT+NNT+ER, slope | 0.16618 | 50 | 0.57762 | 500 |
|  | 0.73322 | 500 | 0.68718 | 600 |
|  | 0.78566 | 550 | 0.71852 | 650 |
|  | 0.80597 | 575 | 0.79206 | 750 |
|  | 0.81980 | 600 | 0.80562 | 775 |
|  | 0.84260 | 650 | 0.82936 | 800 |
|  | 0.87704 | 700 |  |  |
|  | 0.89257 | 750 |  |  |
|  | 0.90123 | 775 | 0.90062 | 1000 |
| ER vs WNUM+TNT+NNT+ARR, slope | 0.75142 | 500 | 0.59957 | 500 |
|  | 0.79251 | 550 | 0.70062 | 600 |
|  | 0.80136 | 575 | 0.80592 | 750 |
|  | 0.87836 | 700 | 0.83510 | 800 |
|  | 0.90142 | 750 | 0.87742 | 900 |
|  |  |  | 0.89800 | 950 |
|  |  |  | 0.91022 | 1000 |

By including about 750-800 subjects in each group, we should be able to reject the null hypotheses.

Additional analysis

Additional questions that this study will address are:

- - Do different presentation formats affect the tenacity of the original decision when baseline risk is altered towards provoking the opposite decision?
  - How do salience and numeracy affect treatment choice?
  - Do different presentations affect espoused understanding of risk reduction presentation, and confidence and satisfaction in decision-making?
  - Are the answers to the above questions consistent across the general public, general practitioners (GPs), and other health professionals?

Conditional logistic regression will be used to see if different presentation formats affect the tenacity of the original decision when baseline risk is altered towards provoking the opposite decision. Salience and numeracy will be included as covariates in the ordinary logistic regression model (Eq 1); one running of the model including both variables at the same time, and one running for each of the covarates (included as one single covariate in the model, according to the proposals of Hosmer and Lemeshow^8^). Chi-square tests will be performed to evaluate the association between risk reduction presentations and confidence, satisfaction in decision-making, as well as consistency across the general public, GPs and other health professionals. If there are too few participants in the various categories, pooling of categories will be done.

Numeracy will be scored as 2, 1 or 0 depending on how many of the following questions the participant answers correctly: ‘How would you express 3 out of 10 as a percentage?’ and ‘If you flip a coin 1000 times, how many times would you expect it to come up with heads?. The salience score can range from zero to 3, table xx Salience score.

# Ethics

This study has been reviewed and approved by the ethical committee of the University of Albany Medical School, Albany, New York. The interventions in this study, alternative ways of presenting information on risk reduction, and asking participants to make a hypothetical decision, are none-invasive and harmless. The same information on reduction of CVD-risk associated with statin use is widely available and is presented in many different ways, including those used in the study.

Participants are informed on the consent screen that they can leave the study at any time, and are given the option of choosing to have any data that they might have entered deleted.

Confidentiality of data entered by participants is ensured by not collecting any information that would make it possible to identify participants. If participants fill out forms to request study results or to participate in the lottery, their email or postal addresses will not be stored in the same database as their responses nor will they these databases be linked in any way.

## REFERENCES

1. McGettigan P, Sly K, O’Connell D, Hill S, Henry D. The effects of information framing on the practices of physicians. J Gen Intern Med. 1999; 14:633-42.
2. Herrin J, Schünemann H, Oxman AD, Vist G, Olsen K. Presentation of empirical evidence about health (Cochrane Review). In: The Cochrane Library. Oxford: Update Software. Under revision.
3. Carling C. et al. Pilot study.
4. Ebrahim S, Davey Smith G, McCabe C, Payne N, Pickin M, Sheldon T A, et al. What role for statins: a review and economic model. Health Technology Assessment 1999, 1-91.
5. Von Neumann J,Morgenstern O. Theory of Games and Economic Behavior. New York: Wiley, 1944.
6. Skolbekken JA. Communicating the risk reduction achieved by cholesterol reducing drugs. BMJ 1998; 316:1956-8.
7. Hollnagel H. On the language of risk in the medical consultation [Danish]. Practicus 1996; 116:237-9.
8. Hosmer David W, Lemeshow Stanley. Applied Logistic Regression, Second edition. John Wiley & Sons, Inc. 2002.

**Table 1 R**isk presentation

| **Presentation  number** |  | **A. Normal risk presentation** | **B. Increased risk presentation** | **C. Decreased risk presentation** |
| --- | --- | --- | --- | --- |
| **1** | **RRR** | Among those who take the pills, there will be a 33% reduced risk of heart disease during the next 10 years. | Among those who take the pills, there will be a 33% reduced risk of heart disease during the next 10 years. | Among those who take the pills, there will be a 33% reduced risk of heart disease during the next 10 years. |
| **2** | **ARR** | Among those who take the pills, there will be a 2% absolute reduction in the risk of getting heart disease during the next 10 years. | Among those who take the pills, there will be an 8% absolute reduction in the risk of getting heart disease during the next 10 years. | Among those who take the pills, there will be a 0.34% absolute reduction in the risk of getting heart disease during the next 10 years. |
| **3** | **NNT** | Among 50 people who take the pills for the next 10 years, there will be one additional person who will not get heart disease during that time. | Among 13 people who take the pills for the next 10 years, there will be one additional person who will not get heart disease during that time. | Among 294 people who take the pills for the next 10 years, there will be one additional person who will not get heart disease during that time. |
| **4** | **Event rates** | Among those who take the pills, the risk of getting heart disease during the next 10 years will be reduced from 6% to 4%. | Among those who take the pills, the risk of getting heart disease during the next 10 years will be reduced from 24% to 16%. | Among those who take the pills, the risk of getting heart disease during the next 10 years will be reduced from 1% to 0.66%. |
| **5** | **TNT** | Among 50 people that take the pills for the next 10 years, they will swallow a total of 182,500 pills and there will be one additional person who will not get heart disease during that time. | Among 13 people that take the pills for the next 10 years, they will swallow a total of 47,450 pills and there will be one additional person who will not get heart disease during that time. | Among 294 people that take the pills for the next 10 years, they will swallow a total of 1,073,100 pills and there will be one additional person who will not get heart disease during that time. |

| **6** | **Whole numbers** | Among 100 people that do not take the pills, 94 will not get heart disease and 6 will get heart disease during the next 10 years. It is not possible to say whether you would be one of the 94 or one of the 6. Among 100 people that do take the pills, 96 will not get heart disease and 4 will get heart disease during the next 10 years. Again, it is not possible to say whether you would be one of the 96 or one of the 4. | Among 100 people that do not take the pills, 76 will not get heart disease and 24 will get heart disease during the next 10 years. It is not possible to say whether you would be one of the 76 or one of the 24. Among 100 people that do take the pills, 84 will not get heart disease and 16 will get heart disease during the next 10 years. Again, it is not possible to say whether you would be one of the 84 or one of the 16. | Among 1000 people that do not take the pills, 990 will not get heart disease and 10 will get heart disease during the next 10 years. It is not possible to say whether you would be one of the 990 or one of the 10. Among 1000 people that do take the pills, 993 will not get heart disease and 7 will get heart disease during the next 10 years. Again, it is not possible to say whether you would be one of the 993 or one of the 7. |
| --- | --- | --- | --- | --- |

**Table 2 Risk levels**

**RISK LEVELS**

**First decision**

| **1st presentation** | **Take pills?** | **2ond presentation** |
| --- | --- | --- |
| Pp = 0.04  Pn = 0.06 | Yes | (decreased risk)  Pp = 0.0066  Pn = 0.01 |
| Pp = 0.04  Pn = 0.06 | NO | (increased risk)  Pp = 0.16  Pn = 0.24 |

Pp = probability of heart disease\taking pills

Pn = probability of heart disease\not taking pills

**Table 3 Salience score**

| Question | Answer | Recoding to use for the analysis |
| --- | --- | --- |
| What do you know about your own cholesterol level? | Measured and OK | 1 |
|  | Measured and high | 2 |
|  | Measured and take medication | 2 |
|  | Measured but do not remember level | 0 |
|  | Don not remember if measured | 0 |
|  | Never measured | 0 |
| Has your doctor told you that you have a heart disease? | Yes | 2 |
|  | No | 0 |
| Do you know someone who has experienced one of the following? | Angina | If yes to one or more of these alternatives: 1  Else 0 |
|  | A mild heart attack |  |
|  | A severe heart attack |  |
|  | Death from a heart attack |  |
|  | Heart problem, but unsure what kind |  |
|  |  | The sum = salience score |
