# Supplementary figures and images for: The Effect of Alternative Summary Statistics for Communicating Risk Reduction on Decisions about Taking Statins: A Randomized Trial
Source: PLoS Med. 2009 Aug 25;6(8):e1000134. doi: 10.1371/journal.pmed.1000134 (PMC2724738; doi:10.1371/journal.pmed.1000134)

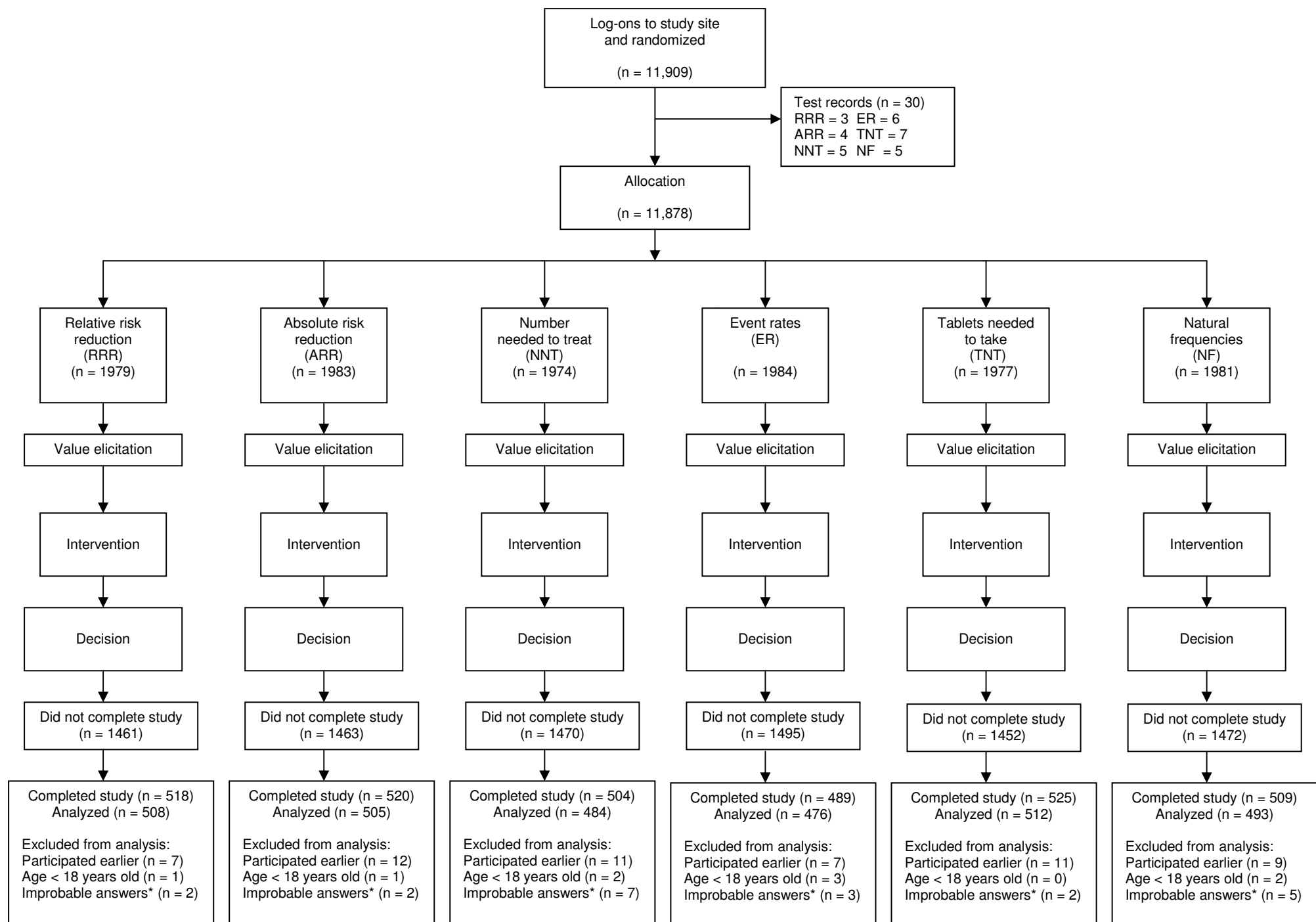

\* The disutility of CHD rated as zero

Supplement: Text S3 — CONSORT flow chart. (0.01 MB PDF) [file pmed.1000134.s003.pdf]
