# Supplementary material for: The Effect of Alternative Summary Statistics for Communicating Risk Reduction on Decisions about Taking Statins: A Randomized Trial
Source: PLoS Med. 2009 Aug 25;6(8):e1000134. doi: 10.1371/journal.pmed.1000134 (PMC2724738; doi:10.1371/journal.pmed.1000134)
Supplement: Text S4 — Risk presentation. (0.01 MB PDF) [file pmed.1000134.s004.pdf]

	Presentation
**Relative risk reduction (RRR)**	Among those who take the pills, there will be a 33% reduced risk of heart disease during the next 10 years.
**Absolute risk reduction (ARR)**	Among those who take the pills, there will be a 2% absolute reduction in the risk of getting heart disease during the next 10 years.
**Number needed to treat (NNT)**	Among 50 people who take the pills for the next 10 years, there will be one additional person who will not get heart disease during that time.
**Event rates (ER)**	Among those who take the pills, the risk of getting heart disease during the next 10 years will be reduced from 6% to 4%.
**Tablets needed to take (TNT)**	Among 50 people that take the pills for the next 10 years, they will swallow a total of 182,500 pills and there will be one additional person who will not get heart disease during that time.
**Natural frequencies (NF)**	Among 100 people that do not take the pills, 94 will not get heart disease and 6 will get heart disease during the next 10 years. It is not possible to say whether you would be one of the 94 or one of the 6. Among 100 people that do take the pills, 96 will not get heart disease and 4 will get heart disease during the next 10 years. Again, it is not possible to say whether you would be one of the 96 or one of the 4.
